# Rapid, High-resolution and Distortion-free $R_{2}^{*}$ Mapping of Fetal Brain using Multi-echo Radial FLASH and Model-based Reconstruction*

**Published:** 2025-01-07

**Authors:** Xiaoqing Wang, Hongli Fan, Zhengguo Tan, Serge Vasylechko, Edward Yang, Ryne Didier, Onur Afacan, Martin Uecker, Simon K. Warfield, Ali Gholipour

**Affiliations:** 1 Department of Radiology, Boston Children’s Hospital, Harvard Medical School, Boston, Massachusetts, USA; 2 Siemens Medical Solutions, Boston, Massachusetts, USA; 3 Department of Radiology, University of Michigan, Ann Arbor, Michigan, USA; 4 Institute of Biomedical Imaging, Graz University of Technology, Graz, Austria; 5 Department of Radiological Sciences, University of California Irvine, Irvine, California, USA; 6 Department of Electrical Engineering and Computer Science, University of California Irvine, Irvine, California, USA

**Keywords:** *R*_2_^*^ mapping, fetal MRI, distortion-free, multi-echo radial FLASH, model-based reconstruction

## Abstract

**Purpose:**

To develop a rapid, high-resolution and distortion-free quantitative $R_{2}^{*}$ mapping technique for fetal brain at 3 T.

**Methods:**

A 2D multi-echo radial FLASH sequence with blip gradients is adapted for fetal brain data acquisition during maternal free breathing at 3 T. A calibrationless model-based reconstruction with sparsity constraints is developed to jointly estimate water, fat, $R_{2}^{*}$ and $B_{0}$ field maps directly from the acquired k-space data. Validations have been performed on numerical and NIST phantoms and five fetal subjects ranging from 27 weeks to 36 weeks gestation age.

**Results:**

Both numerical and experimental phantom studies confirm good accuracy and precision of the proposed method. In fetal studies, both the parallel imaging compressed sensing (PICS) technique with a Graph Cut algorithm and the model-based approach proved effective for parameter quantification, with the latter providing enhanced image details. Compared to commonly used multi-echo EPI approaches, the proposed radial technique shows improved spatial resolution (1.1 × 1.1 × 3 mm^3^ vs. 2–3 × 2–3 × 3 mm^3^) and reduced distortion. Quantitative $R_{2}^{*}$ results confirm good agreement between the two acquisition strategies. Additionally, high-resolution, distortion-free $R_{2}^{*}$-weighted images can be synthesized, offering complementary information to HASTE.

**Conclusion:**

This work demonstrates the feasibility of radial acquisition for motion-robust quantitative $R_{2}^{*}$ mapping of the fetal brain. This proposed multi-echo radial FLASH, combined with calibrationless model-based reconstruction, achieves accurate, distortion-free fetal brain $R_{2}^{*}$ mapping at a nominal resolution of 1.1 × 1.1 × 3 mm^3^ within 2 seconds.

## 1 Introduction

Quantitative $R_{2}^{*}$ (where $R_{2}^{*}=1/T_{2}^{*}$) mapping of the fetal brain is of increasing value. For example, changes in $R_{2}^{*}$ values across gestational age provide a quantitative measure of early brain development [1]. Furthermore, $R_{2}^{*}$ mapping and $R_{2}^{*}$-weighted imaging are valuable in identifying intracranial hemorrhage in the fetal brain [2, 3]. The quantitative values are also playing an important role for optimizing $R_{2}^{*}$-weighted functional fetal MRI [4–6]. However, obtaining accurate and high-resolution $R_{2}^{*}$ imaging of the fetal brain is challenging due to motion caused by maternal respiration and unpredictable fetal movements [7, 8]. As a result, single-shot sequences, particularly single-shot 2D multi-echo Echo-Planar Imaging (EPI)-based approaches [1, 5, 9–12], are typically used for $R_{2}^{*}$ quantification of fetal brain. These techniques were initially developed for 1.5 T [1] and 3.0 T [5, 9], with recent adaptations for 0.55 T [13]. While relatively higher resolution and signal-to-noise ratio (SNR) imaging is achievable at higher fields, low field (e.g., 0.55 T) imaging has shown reduced distortion artifacts for the EPI readout [14], which is attributed to reduced field inhomogeneity and smaller $R_{2}^{*}s$ (i.e., longer $T_{2}^{*}s$). Consequently, quantitative $R_{2}^{*}$ mapping of fetal body organs has also been reported at 0.55 T [15].

Despite the scan efficiency of EPI, its prolonged readout makes EPI susceptible to geometric distortion caused by $B_{0}$ field inhomogeneity, particularly at higher field strengths. Additionally, the extended readout time necessitates a trade-off between imaging speed (short echo times) and spatial resolution due to $T_{2}^{*}$ decay in the multi-echo EPI sequence [16]. For instance, the commonly reported spatial resolution for fetal brain imaging is 3 × 3 × 3 mm^3^ [5, 9, 11], which may limit its usefulness in clinical diagnosis where high-resolution imaging is required [2, 3, 8, 17].

Radial acquisition is an alternative sampling strategy that has gained significant interest in the past decade due to its tolerance to data undersampling and robustness against motion [18–21]. It has been applied to imaging children with reduced sedation [22–24] and in free-breathing fetal studies [25–27]. Stack-of-stars multi-echo radial fast low-angle shot (FLASH) sequence has also been used for quantitative $R_{2}^{*}$ mapping in adult abdominal imaging [28–31] and the fetal placenta [27]. However, the unpredictable motion of the fetal brain, combined with maternal motion and motion-induced phase errors, poses significant challenges for applying 3D sequences to quantitative imaging of the fetal brain.

Alongside motion-robust sequence design, advanced image reconstruction is essential for efficient quantitative imaging. Reconstruction techniques that incorporate prior signal model information to constrain parameter space have been developed [32–38]. Among these, nonlinear model-based reconstruction techniques [38, 39] are highly efficient. These techniques incorporate complex spin dynamics directly in the reconstruction. By formulating reconstruction as a nonlinear inverse problem, model-based reconstruction can estimate physical quantitative maps from undersampled k-space data without intermediate reconstruction or pixelwise fitting. Advanced regularization techniques, such as sparsity constraints [40], further enhance precision in quantitative mapping. Recently, this approach has been extended to reconstruct water, fat, and $R_{2}^{*}$ maps from undersampled 3D multi-echo FLASH for liver imaging [28, 41], also enabling additional $B_{0}$ estimation [30].

Building on the ideas above, this work aims to develop a rapid quantitative $R_{2}^{*}$ mapping of fetal brain utilizing a 2D multi-echo radial FLASH sequence with blip gradients and a calibrationless nonlinear model-based reconstruction. While the radial sequence provides motion robustness and efficient k-space coverage for fetal imaging, the model-based reconstruction estimates quantitative maps directly from undersampled k-space, reducing the number of unknowns in the estimation process. This combination enables high-resolution and distortion-free quantitative $R_{2}^{*}$ mapping (1.1 × 1.1 × 3 mm^3^) of fetal brain in **two seconds per slice**. Validations have been performed on numerical simulations, experimental phantom, and five fetuses each scanned at an age between 27 to 36 weeks of gestation.

## Methods

### Sequence Design

A 2D multi-echo radial FLASH sequence is adapted for data acquisition. Similar to [42], radial spokes are designed to rotate along the echo dimension using blip gradients, enabling an efficient k-space coverage (Supporting Information Figure S1). The distribution of spokes is designed in a way that radial lines from several excitation (e.g., 3) and all echoes are equally distributed [42] in one k-space, with an angle $\theta_{l,m}=2\pi /\left(N_{E}\cdot N_{S}\right)\cdot \left[(l-1)\cdot N_{E}+m-1\right]$ for the l th TR and the m th echo. $N_{E}$ and $N_{S}$ are the number of echoes and shots (TRs) per k-space, respectively. Spokes acquired in consecutive k-space frame are then rotated by a small golden-angle (≈ 68.75°) with respect to the previous one [43] to enable a complementary coverage of k-space. Since the $R_{2}^{*}$ values of fetal brain are reported to be much smaller than those of adult brains [4], the number of echoes is extended from 7 [44] to 35 to enable a robust $R_{2}^{*}$ estimation.

### Signal Equation and Model-based Reconstruction

Although the fetal brain contains minimal fat, surrounding tissues, such as maternal body tissue, include fat. To account for this, we construct the signal equation as follows [30]:

(1) $$M_{{TE}_{m}}=\left(W+F\cdot z_{m}\right)\cdot exp\left({TE}_{m}\cdot i2\pi \cdot f_{B_{0}}\right)\cdot exp\left(-{TE}_{m}\cdot R_{2}^{*}\right)$$

with W and F being the water and fat components, respectively; $z_{m}$ is the summarized 6-peak fat spectrum [45] at echo time ${TE}_{m}$; and $f_{B_{0}}$ and $R_{2}^{*}$ are the corresponding field map and relaxation rate, respectively. The estimation of the unknowns ${\left(W,F,R_{2}^{*},f_{B_{0}}\right)}^{T}$ is then formulated as a nonlinear inverse problem; i.e., by combining the above physical model with the parallel imaging equation [46, 47], we construct a nonlinear forward operator F, which maps the unknowns in Equation (1) and the unknown coil sensitivities C to the acquired multi-channel data y at ${TE}_{m}$, i.e.,

(2) $$F:x\mapsto y=𝒫ℱC\cdot M_{{TE}_{m}}\left(x_{p}\right).$$

Here, 𝒫 is the sampling pattern and ℱ is the Fourier transform. By defining $x_{c}={\left(c_{1},\ldots ,c_{k},\ldots ,c_{K}\right)}^{T}$, with $c_{k}$ the individual k th coil sensitivity map, the vector of unknowns in Equation (2) is $x={\left(x_{p},x_{c}\right)}^{T}$. The estimation of x is then formulated as an optimization problem, i.e.,

(3) $$\overset{ˆ}{x}={argmin}_{x\in D}\frac{1}{2}\sum_{TE}\;{\left\|PℱC\cdot M_{{TE}_{m}}(x)-Y_{{TE}_{m}}\right\|}_{2}^{2}+R(x).$$

Here, D is a convex set, ensuring non-negativity of $R_{2}^{*}.\;R(\cdot )$ is the regularization term for both parameter maps and coil sensitivity maps. In particular, we use joint $\ell_{1}$-Wavelet sparsity constraint [48] on ${\left(W,F,R_{2}^{*}\right)}^{T}$ to exploit sparsity and correlations between maps and Sobolev regularization on the $f_{B_{0}}$ map [30, 44] and the coil sensitivity maps [47] to enforce smoothness. The above optimization problem is solved by IRGNM-FISTA [48] using the Berkeley Advanced Reconstruction Toolbox (BART) [49]. More details for IRGNM-FISTA can be found in Ref. [48].

### Numerical Simulations

To validate the accuracy of the proposed approach, a numerical phantom with ten circular tubes and a background was simulated. The $R_{2}^{*}$ values were set to be from 10 s^−1^ to 200 s^−1^ (i.e., $T_{2}^{*}$ from 10 ms to 200 ms with a step size of 20 ms). The off-resonance ranged from −50 Hz to 50 Hz with a step size of 10 Hz. The fat fraction was set to be 20% for all tubes and backgrounds. The k-space data was derived from the analytical Fourier representation of an ellipse assuming an array of eight circular receiver coils surrounding the phantom. The 2D multi-echo radial FLASH sequence described in the sequence design section was used to sample the simulated k-space with a base resolution of 192 pixels covering a field of view of 128 mm. The other sequence parameters are the same as those listed in the following Experiments section. Complex white Gaussian noise with a standard deviation of 0.1 was added to the simulated k-space data.

### Experiments

All MRI experiments were conducted on a Magnetom Prisma 3T scanner (Siemens Healthineers, Erlangen, Germany) during maternal free breathing. The study was approved by the Institutional Review Board, and written informed consent was obtained from all participants. Validation was first performed using the $T_{1}$ spheres of a NIST phantom [50]. Phantom scans employed a 64-channel head/neck coil, while fetal imaging utilized a 30-channel abdominal coil. Five pregnant female subjects (29 ± 7 years old; fetuses: 32.6 ± 3.6 weeks) without known illness were enrolled and scanned. Standard Half Fourier Single-shot Turbo spin-Echo (HASTE) images were acquired first for each subject in three (axial, coronal, and sagittal) orientations with a FOV of 256 × 256 mm^2^, matrix size= 256 × 256, slice thickness = 2 mm, and a total acquisition time of 1–1.5 second per slice. Radial fetal scans were performed with the following acquisition parameters: FOV = 256 × 256 mm^2^, matrix size= 224 × 224, slice thickness = 3 mm, 35 echoes with TR = 68.3 ms, TE_1_/δTE/TE_35_ = 2.37/1.88/66.90 ms, FA = 20°, bandwidth = 740 Hz/pixel, and 30 RF excitations with 1050 radial acquired spokes for all echoes. The multi-echo EPI images were acquired for quantitative comparison. The parameters for EPI were: FOV = 256 × 256 mm^2^, matrix size= 96–128 × 96–128, slice thickness = 3 mm, TEs = (23.4–29.8, 74.90–77.48, 126.38–147.20, 177.88–207.46) ms. Repeated radial scans were able to be conducted on three subjects to assess repeatability of the proposed method.

Additionally, for the phantom study, a vendor-provided 3D Cartesian multi-echo sequence was used for reference with these parameters: FOV = 256 × 256 mm^2^, matrix size= 224 × 224, slice thickness = 3 mm with 30 slices, 11 echoes with TR = 65 ms, TE_1_/δTE/TE_11_ = 6.0/5.5/61.0 ms, FA = 15°, bandwidth = 300 Hz/pixel, and acceleration factor 2. The total acquisition time was 4:17 min.

### Iterative Reconstruction

All iterative reconstructions were performed offline using BART [49]. The multi-echo radial FLASH datasets from multiple receiver coils were first corrected for gradient delay errors using RING [51] and then compressed to 12 virtual coils via principal component analysis. The data and sampling trajectory were subsequently gridded onto a Cartesian grid, where all iterative steps were carried out using FFT-based convolutions with the point-spread function [52, 53]. The model-based iterative reconstruction was executed on a GPU with 48 GB of memory (RTX A6000, NVIDIA, Santa Clara, CA), with a computation time of 5–10 minutes per dataset. For comparison, the same multi-echo datasets were jointly reconstructed using the parallel imaging and compressed sensing (PICS) method, with coil sensitivity maps estimated from the first echo and joint sparsity constraints applied across spatial and echo dimensions. After image reconstruction, quantitative water, fat, $R_{2}^{*}$, and $B_{0}$ maps were estimated using the Graph Cut technique [54], available in the ISMRM water-fat toolbox [55].

### Quantitative Analysis

All quantitative results are reported as mean ± standard deviation (SD). Regions-of-interest (ROIs) were carefully drawn into the frontal white matter (FWM), thalamic gray matter (THA), and occipital white matter (OWM) regions [1] of the quantitative fetal brain $R_{2}^{*}$ maps using the arrayShow [56] tool implemented in MATLAB (MathWorks, Natick, MA). Bland–Altman analyses were used to compare ROI-based mean quantitative values between reference and the proposed method.

## Results

**Figure 1.**
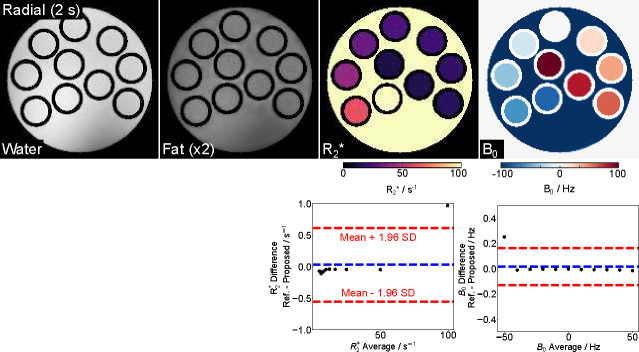
(Top) Model-based estimated water, fat (×2), $R_{2}^{*}$, and $B_{0}$ field maps using a 2-second multi-echo radial FLASH sequence for a numerical phantom. (Bottom) Bland-Altman plots comparing the ROI-analyzed mean quantitative values to the ground truth. The mean differences are 0.03 ± 0.3 s^−1^ and 0.02 ± 0.07 Hz for $R_{2}^{*}$ and $B_{0}$, respectively.

We first validated the proposed technique on a numerical phantom, which offers a broad range of ground-truth quantitative values under noisy conditions. Figure 1 (top) shows water, fat, $R_{2}^{*}$, and $B_{0}$ maps obtained from the model-based reconstruction with a 2-second multi-echo radial FLASH acquisition. Figure 1 (bottom) compares ROI-analyzed quantitative values with the ground truth. The mean differences are 0.03 ± 0.3 *s*^−1^ and 0.02 ± 0.07 Hz for $R_{2}^{*}$, and $B_{0}$, respectively. The low mean differences indicate good quantitative accuracy of the proposed method.

**Figure 2.**
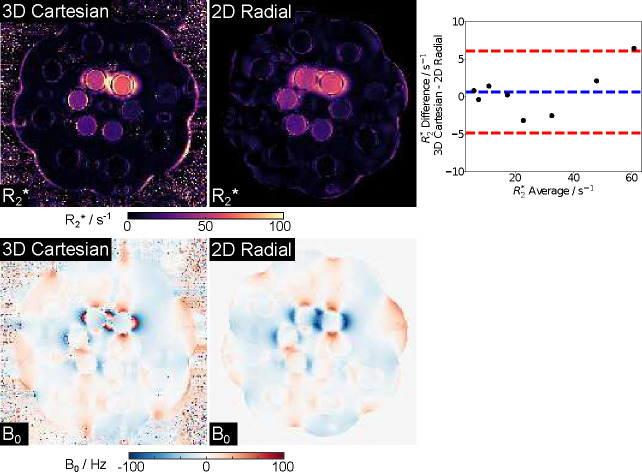
Model-based estimated (top) $R_{2}^{*}$ and (bottom) $B_{0}$ maps and their comparison to the 3D Cartesian references of the NIST phantom (T1 sphere). (Top right) Bland–Altman plots comparing the ROI-analyzed mean quantitative $R_{2}^{*}$ values to the references. The mean difference is 0.6±2.8 s^−1^. Note that the 3D Cartesian reference acquisition time is 4:17 min, while the 2D radial sequence requires 2 seconds per slice.

Figure 2 presents NIST $R_{2}^{*}$ (top) and $B_{0}$ (bottom) maps generated by the proposed method and a 3D Cartesian reference. Note that here a 3-parameter model (i.e., excluding fat in Equation 1) was employed in the reconstruction as there is no known fat component in the NIST phantom. Despite phase wrap differences around the central top two tubes on the $B_{0}$ maps, both visual inspection and quantitative ROI analysis demonstrate good agreement: The mean $R_{2}^{*}$ difference is 0.6 ± 2.4 s^−1^ for $R_{2}^{*}$ ranging from 4 s^−1^ to 60 s^−1^.

**Figure 3.**
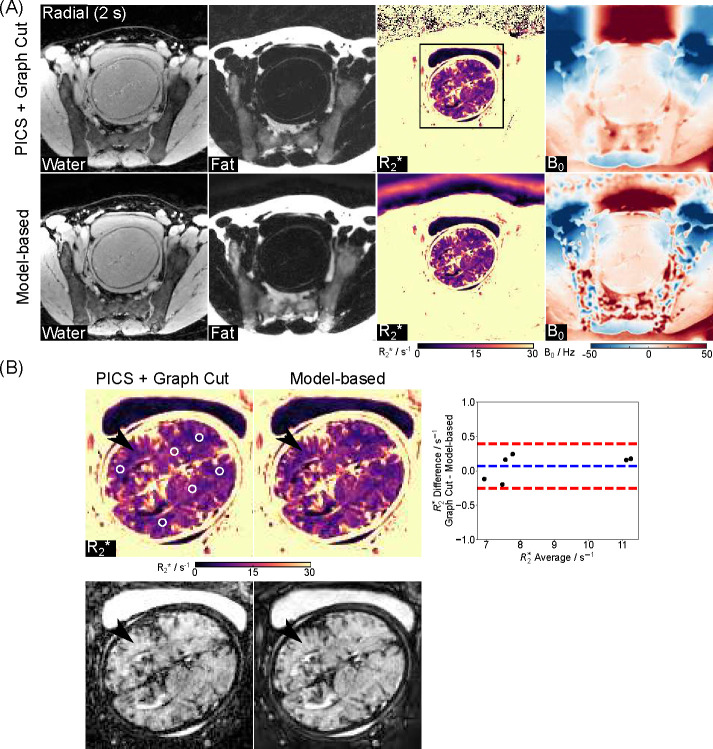
(A). Model-based reconstructed water, fat, $R_{2}^{*}$, and $B_{0}$ maps and their comparison to a reference method (Parallel imaging compressed sensing with Graph Cut) utilizing the same radial data. (B). Enlarged $R_{2}^{*}$ maps and $R_{2}^{*}$-weighted images (TE = 60 ms) and the Bland–Altman plots comparing the ROI-analyzed (white circles) mean quantitative $R_{2}^{*}$ values. The mean difference is 0.07±0.17 s^−1^ for all ROIs. Black arrows indicate a better balance between preserving fine details and reducing noise of the model-based approach.

**Figure 4.**
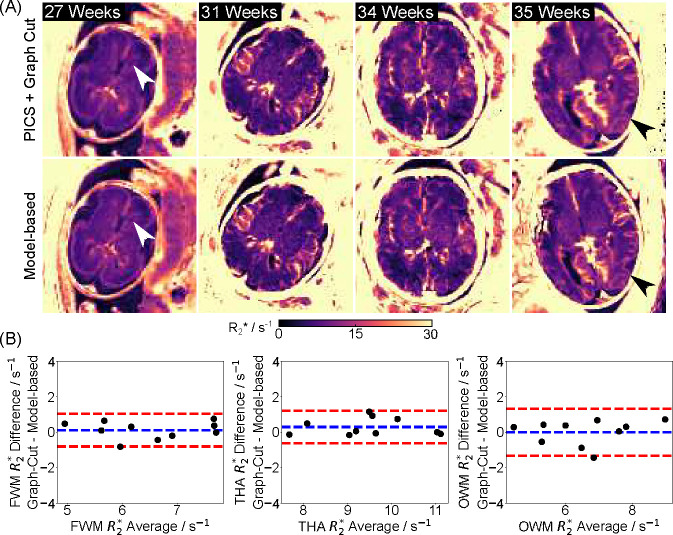
(A). Comparison of quantitative fetal brain $R_{2}^{*}$ maps estimated using (top) PICS with Graph Cut and (bottom) model-based reconstruction for the other four subjects. White and black arrows indicate improved image details by model-based reconstruction. (B). Bland–Altman plots comparing the mean quantitative $R_{2}^{*}$ values for all five subjects. The mean $R_{2}^{*}$ differences for FWM, THA and OWM are 0.12±0.47 s^−1^, 0.30 ± 0.47 s^−1^, and 0.002 ± 0.68 s^−1^, retrospectively.

Figure 3 (A) shows reconstructed water, fat, $R_{2}^{*}$, and $B_{0}$ maps obtained using the proposed model-based method and a PICS reconstruction with the Graph Cut technique on the same radial dataset. Visual inspection indicates good correspondence between the two methods. Figure 3 (B) includes enlarged $R_{2}^{*}$ maps, synthesized $R_{2}^{*}$-weighted images at TE = 60 ms, and Bland-Altman plots comparing mean $R_{2}^{*}$ values for selected ROIs (white circles). Despite the proposed model-based method showing a better balance between preserving fine details and reducing noise in both $R_{2}^{*}$ maps and synthesized $R_{2}^{*}$-weighted images (black arrows), the low mean difference (0.07 ± 0.17 s^−1^) confirms strong quantitative agreement. The above findings are further supported by comparisons across four additional subjects and quantitative results shown in Figure 4. Figure 4 (A) highlights comparable image quality with enhanced details in the model-based reconstruction (white and black arrows), while Figure 4 (B) demonstrates small quantitative differences between the two reconstruction approaches.

**Figure 5.**
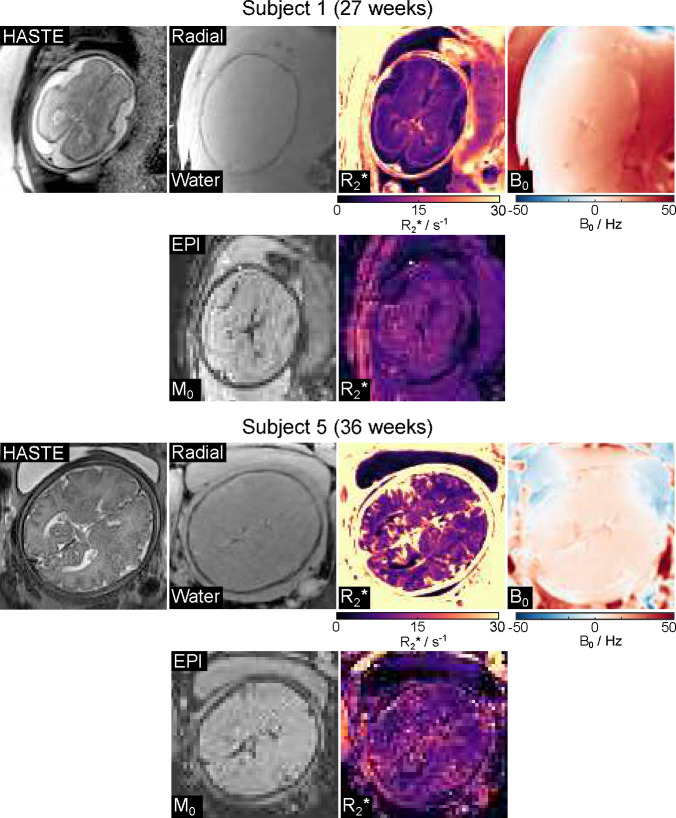
HASTE images, model-based reconstructed water, $R_{2}^{*}$, and $B_{0}$ maps, along with a comparison to the EPI results for two representative subjects (27 weeks, top; 36 weeks, bottom). The radial acquisition demonstrates notably improved spatial resolution and reduced distortions compared to the EPI counterpart in both cases.

**Figure 6.**
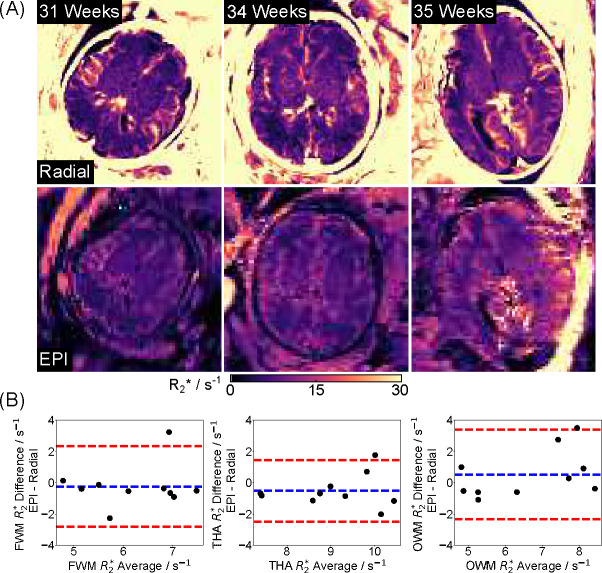
(A). Quantitative $R_{2}^{*}$ maps estimated with (top) multi-echo radial FLASH using model-based reconstruction and (bottom) EPI for the other three subjects. (B). Bland–Altman plots comparing ROI mean quantitative $R_{2}^{*}$ values between the proposed technique and the EPI method for five subjects, showing a mean difference of −0.2 ± 1.3 s^−1^, −0.5 ± 1.0 s^−1^ and 0.5 ± 1.5 s^−1^ for FWM, THA and OWM, respectively.

Figure 5 presents estimated radial water, $R_{2}^{*}$, and $B_{0}$ maps with model-based reconstruction, along with EPI $M_{0}$ and $R_{2}^{*}$ maps and T2-weighted HASTE images for two representative subjects (27 weeks and 36 weeks). Apart from motion-related differences, qualitative assessment demonstrates improved spatial resolution and reduced distortion by the proposed radial technique in both cases. Figure 6 (A) compares quantitative $R_{2}^{*}$ maps generated from the radial and EPI techniques for the other three subjects. Consistent with Figure 5, the radial $R_{2}^{*}$ maps exhibit higher spatial resolution and less distortion compared to EPI maps. Figure 6 (B) presents ROI-analyzed quantitative values for both methods across all five subjects. The mean differences for FWM, THA and OWM are −0.2 ± 1.3 s^−1^, −0.5 ± 1.0 s^−1^, and 0.5 ± 1.5 s^−1^ between radial and EPI approaches. Additionally, Table 1 presents the mean $R_{2}^{*}$ values for all subjects. The EPI mean $R_{2}^{*}$ values are 6.1±1.2 s^−1^, 8.8±1.3 s^−1^, and 6.9±1.9 s^−1^, while the radial ones are 6.4±1.0 s^−1^, 9.3±1.1 s^−1^, 6.4±1.3 s^−1^ for FWM, THA and OWM, respectively.

**Figure 7.**
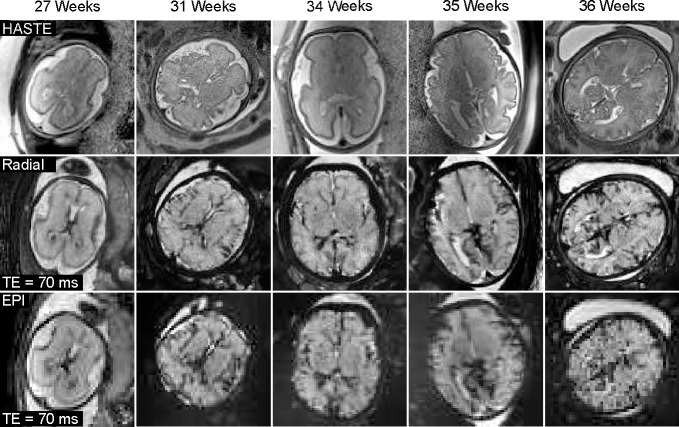
(Top) HASTE images, synthesized $R_{2}^{*}$-weighted images at TE = 70 ms for (middle) radial and (bottom) EPI acquisitions. The nominal spatial resolution for HASTE, radial FLASH and EPI are 1.0 × 1.0 × 2 mm^3^, 1.1 × 1.1 × 3 mm^3^, and 2–3 × 2–3 × 3 mm^3^, respectively.

Supporting Information Figure S2 (A) presents two repetitive fetal brain $R_{2}^{*}$ maps for three subjects. Despite varying motion conditions, the quantitative maps appear visually consistent. This observation is confirmed by the minimal quantitative differences observed in the selected ROIs, as shown in Supporting Information Figure S2 (B). In addition to quantitative maps, Figure 7 demonstrates synthesized $R_{2}^{*}$-weighted images at TE= 70 ms (a typical value chosen for fetal functional MRI study) of the proposed radial approach and EPI methods as well as T2-weighted HASTE images across all subjects. In line with $R_{2}^{*}$ images, the contrast-weighted radial images demonstrate improved spatial resolution and reduced distortion compared to EPI. Moreover, the radial FLASH images are less affected by the B1 inhomogeneity than the T2-weighted HASTE images, which could provide additional values for fetal imaging.

The supporting information videos S1 and S2 provide the estimated radial $R_{2}^{*}$ maps and corresponding synthesized $R_{2}^{*}$-weighted images at TE = 70 ms for 20 slices of the same subjects shown in Figure 5 (i.e., Subject 1: 27 weeks, Subject 5: 36 weeks). The datasets were acquired in a slice-interleaved manner and reordered for video formatting. While S1 demonstrates the proposed radial approach can produce high-resolution $R_{2}^{*}$ maps and contrast-weighted images for a fetal brain with rapid motion, S2 shows high-resolution $R_{2}^{*}$ maps and images can be readily achieved by the proposed approach when the fetal brain remains more stable.

## Discussion

In this work, we present a rapid, high-resolution, and distortion-free $R_{2}^{*}$ mapping technique for the fetal brain. With the multi-echo radial sequence offering motion robustness and efficient k-space coverage, the regularized calibrationless model-based reconstruction efficiently estimates quantitative maps and coil sensitivity maps directly from undersampled k-space data. Validation through simulations, phantom studies, and data from five fetal subjects confirms reliable and accurate $R_{2}^{*}$ measurements compared to reference methods. This proposed approach achieves distortion-free $R_{2}^{*}$ mapping of fetal brain at a nominal resolution of 1.1 × 1.1 × 3 mm^3^ within 2 seconds. Furthermore, the developed method enables the generation of high-resolution $R_{2}^{*}$-weighted images, offering complementary information to the conventional T2-weighted HASTE images for fetal imaging.

To the best of our knowledge, this is the first study utilizing motion-robust 2D radial acquisition for rapid, high-resolution, and distortion-free $R_{2}^{*}$ mapping of the fetal brain. The proposed radial method shows improved spatial resolution and reduced distortion than the conventional multi-echo EPI approaches for all cases. Despite differences in fetal position, spatial resolution and distortions between these two acquisition strategies, quantitative results demonstrate a good agreement between the two. Moreover, our results indicate both the PICS with Graph Cut and model-based reconstruction are effective for parameter quantification. Although PICS with graph cut is considered the state-of-the-art method for quantification of $R_{2}^{*}$ and $B_{0}$ in body imaging, the model-based approach offers enhanced image details by directly reconstructing parameter maps from k-space with direct regularization applied to quantitative $R_{2}^{*}$ maps. Compared to the values in the literature, the present $R_{2}^{*}$ values (both radial and EPI) are slightly higher, especially for the THA regions. This could be due to the age difference in the studied fetal groups as $R_{2}^{*}$ values change rapidly along the gestation age. A more detailed analysis of $R_{2}^{*}$ variation across age and between subjects warrants a larger scale study, which requires enrolling and scanning a larger number of subjects.

Stack-of-stars radial multi-echo acquisitions [21] have been employed for 3D $R_{2}^{*}$ mapping of the placenta [27], and our previous work extended this approach to fetal brain $R_{2}^{*}$ mapping [57]. While these 3D methods perform well for fetal brains with minimal or no motion, they require extended acquisition times (over 3 minutes), posing challenges in cases of rapid fetal brain motion, even with advanced motion correction techniques. In contrast, the proposed 2D technique delivers reliable $R_{2}^{*}$ maps within a short acquisition window, demonstrating robustness in scenarios with significant fetal motion. Moreover, the proposed method is very general and can be extended to the quantification of other challenging fetal organs. For instance, high-resolution quantitative $R_{2}^{*}$ mapping of the fetal liver is of great interest as it could provide valuable insights into evaluating liver iron overload in the fetal stage.

As a technical development study, this work is limited by the relatively small number of subjects. Moreover, repeatability could only be assessed for 3 out of 5 subjects due to limited scan time during the development phase. Future studies will apply the technique to a larger cohort to investigate brain development, focusing on how $R_{2}^{*}$ values change across gestational ages. Repeated scans will also be conducted on more subjects to comprehensively evaluate the technique’s repeatability. Furthermore, the 2-second acquisition time, while effective, remains longer than the HASTE sequence. The latter typically takes less than 1 second and is highly effective at freezing motion. Consequently, although the proposed radial acquisition is robust to motion, this method may still be affected by very rapid motion during data acquisition. Our retrospective analysis of Subject 5 (Supporting Information Figure S3) demonstrates that the proposed method can produce reasonable images within 1 second, albeit with increased noise and reduced $R_{2}^{*}$ accuracy. To address this, future work will focus on further reducing acquisition time without compromising accuracy or precision. One potential approach would be to replace the hand-crafted $\ell_{1}$-Wavelet transform with a deep-learning-enhanced regularizer [58] in the model-based reconstruction. Another idea would be to adapt radial simultaneous multi-slice techniques [59] for sub-second quantitative fetal brain imaging.

## Conclusion

This work demonstrates the feasibility of radial acquisition for motion-robust quantitative $R_{2}^{*}$ mapping of the fetal brain. By combining multi-echo radial FLASH with calibrationless model-based reconstruction, the proposed method achieves accurate, distortion-free fetal brain $R_{2}^{*}$ mapping at a nominal resolution of 1.1 × 1.1 × 3 mm^3^ within 2 seconds.

## Supplementary Material

Supplement 1

## Figures and Tables

**Table 1:** Quantitative $R_{2}^{*}$ values (s^−1^, mean ± SD) for fetal brains.

Tissue	FWM	THA	OWM

Radial 3T	6.4 ± 1.0	9.3 ± 1.1	6.4 ± 1.3
EPI 3T	6.1 ± 1.2	8.8 ± 1.3	6.9 ± 1.9
Rivkin et al.[4] 1.5 T	6.6	7.9	
Vasylechko et al.[1] 1.5 T	4.3	6.5	4.0
Blazejewska et al.[5] 1.5 T	3.9	6.0	
Vasylechko et al.[9] 3.0 T	5.0	6.6	4.4

## Data Availability

In the spirit of reproducible research, code and data to reproduce the reconstruction and analysis in this work will be available on https://github.com/IntelligentImaging/FetalR2Star.
