# Supplementary material for: Rapid, High-resolution and Distortion-free R2* Mapping of Fetal Brain using Multi-echo Radial FLASH and Model-based Reconstruction
Source: ArXiv. 2025 May 27:arXiv:2501.00256v4. Preprint. [Version 4] (PMC11722525)
Supplement: 1 [file NIHPP2501.00256V4-supplement-1.pdf]

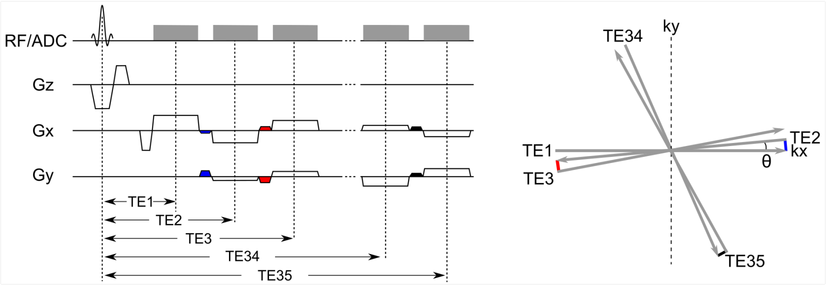

Supporting Information Figure S1. Schematic diagram of the 2D multi-echo radial FLASH sequence (the first three echoes and the last two echoes are shown). Blip gradients (blue, red and black regions) are introduced among echoes to enable a complementary k-space coverage. θ is determined in a way that spokes from all echoes and 3 TRs are equally distributed. I.e., with 35 echoes and 3 TRs, $\theta = 360^\circ/(35 \times 3)$.

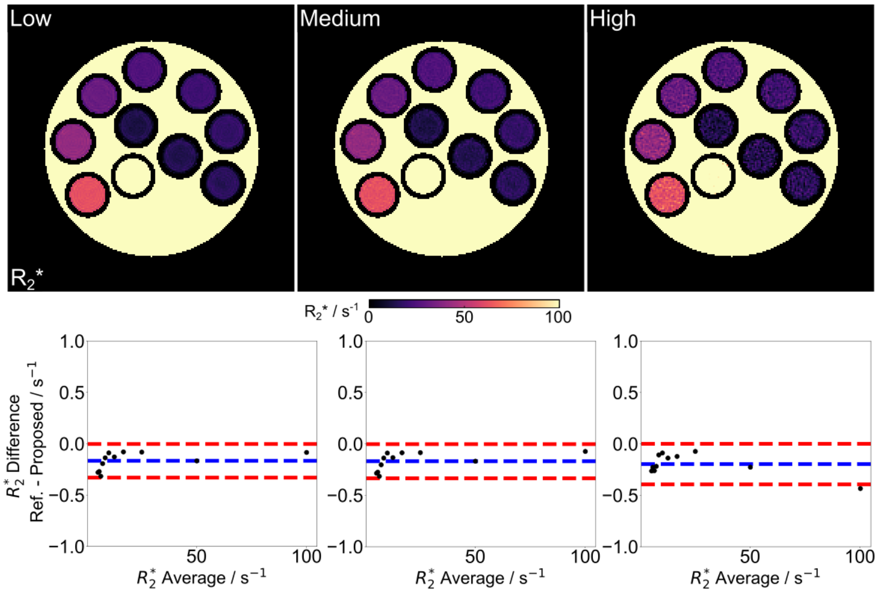

Supporting Information Figure S2. (Top) Quantitative R_2^* maps estimated from simulated data using the proposed model-based approach under (left) low, (middle) medium, and (right) high levels of Gaussian noise. (Bottom) Bland–Altman plots comparing ROI-based R_2^* estimates between the proposed method and ground truth. The mean differences are $-0.17 \pm 0.06\ s^{-1}$, $-0.17 \pm 0.07\ s^{-1}$, and $-0.18 \pm 0.07\ s^{-1}$, respectively. Detailed quantitative values for each tube (mean $\pm$ standard deviation) are provided in Supporting Information Table S1.

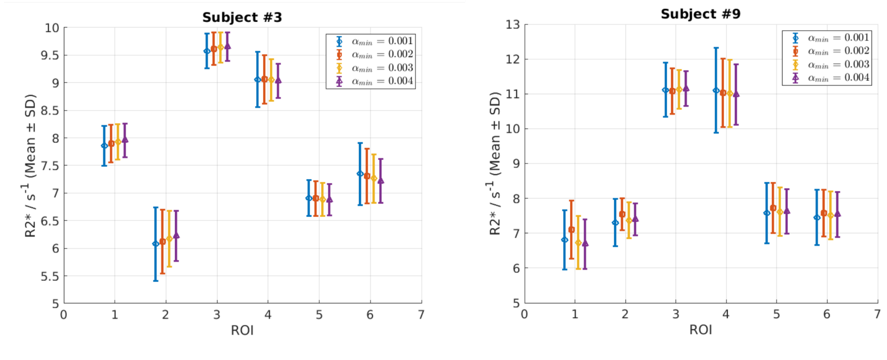

Supporting Information Figure S3. Quantitative R_2^* values (mean and standard deviation) within ROIs that were manually drawn into the frontal white matter, thalamic gray matter, and occipital white matter regions of all R_2^* maps in Figure 3.

Table 1: Supporting Information Table S1. Quantitative R_2^* values (s^{-1}, mean $\pm$ SD) for the numerical phantom with different noise levels in the Supporting Information Figure S2.

True T_2^* / ms	True R_2^* / s^{-1}	Estimated R_2^* / s^{-1} across noise levels		
		Low	Medium	High
10	100	100.2 ± 2.1	100.2 ± 2.3	100.2 ± 7.3
20	50	50.1 ± 0.9	50.1 ± 1.4	50.2 ± 6.2
40	25	25.1 ± 0.6	25.1 ± 0.8	25.1 ± 2.6
60	16.7	16.7 ± 0.6	16.8 ± 0.9	16.8 ± 2.7
80	12.5	12.6 ± 0.6	12.6 ± 0.8	12.6 ± 2.5
100	10	10.1 ± 0.6	10.1 ± 0.8	10.1 ± 2.2
120	8.3	8.5 ± 0.6	8.5 ± 0.8	8.4 ± 2.4
140	7.1	7.3 ± 0.6	7.3 ± 0.9	7.4 ± 2.5
160	6.3	6.6 ± 0.7	6.6 ± 0.9	6.5 ± 2.7
180	5.6	5.8 ± 0.8	5.8 ± 0.9	5.8 ± 2.9
200	5.0	5.2 ± 0.7	5.2 ± 0.9	5.3 ± 2.5

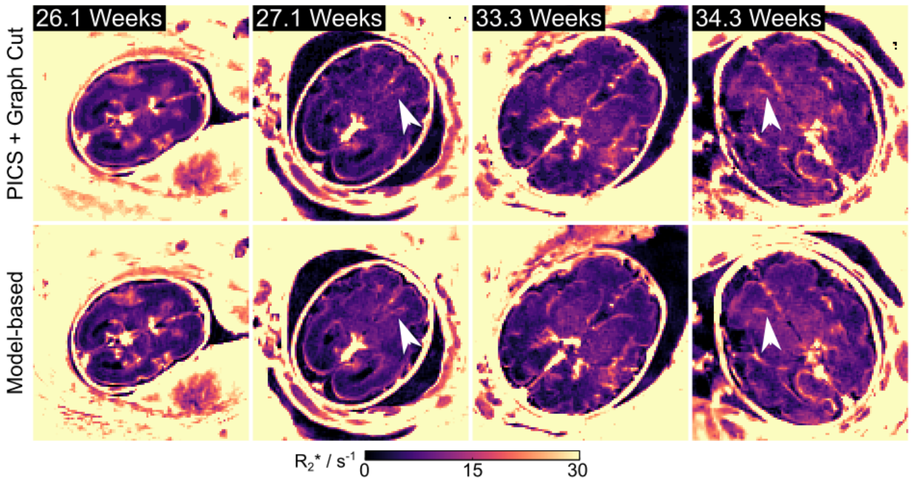

Supporting Information Figure S4. Comparison of quantitative fetal brain R_2^* maps estimated using (top) PICS with Graph Cut and (bottom) model-based reconstruction for the remaining four subjects at different gestational ages. Similar to Figure 5 (A), white arrows indicate improved image details by model-based reconstruction. Quantitative comparison of all subjects is presented in Figure 5 (B).

Table 2: Quantitative R_2^* values (s^{-1}, mean ± SD) for fetal brains.

Tissue	FWM	THA	OWM
Radial 3T (Model-based)	6.1 ± 1.0	9.1 ± 1.3	6.0 ± 1.3
Radial 3T (PICS + Graph Cut)	6.2 ± 1.0	9.2 ± 1.4	5.9 ± 1.5
EPI 3T	5.7 ± 1.1	8.7 ± 1.1	6.4 ± 1.6
Rivkin et al.[4] 1.5 T	6.6	7.9	
Vasylechko et al.[1] 1.5 T	4.3	6.5	4.0
Blazejewska et al.[5] 1.5 T	3.9	6.0	
Vasylechko et al.[9] 3.0 T	5.0	6.6	4.4

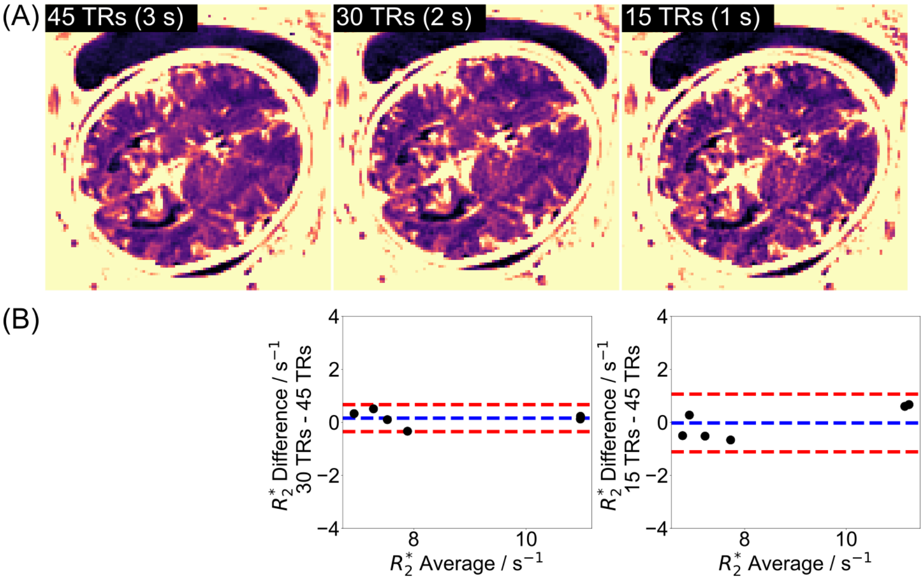

Supporting Information Figure S5. (A). Quantitative R_2^* maps estimated using 3-second (45 TRs), 2-second (30 TRs), and 1-second (15 TRs) multi-echo radial FLASH acquisitions for Subject 9. Because of minimal motion during data acquisition, the 2-second and 1-second data were retrospectively undersampled from the 3-second one. (B) Bland-Altman plots comparing mean R_2^* values between the 3-second and 2-second, and 3-second and 1-second acquisitions. The mean R_2^* differences are 0.16 ± 0.26 s^{-1} and -0.02 ± 0.6 s^{-1}, respectively.

Supporting Information Video S1. Quantitative R_2^* maps and synthesized R_2^*-weighted images (TE = 70 ms) for 16 slices of Subject 3 (27.9 weeks).

Supporting Information Video S2. Quantitative R_2^* maps and synthesized R_2^*-weighted images (TE = 70 ms) for 20 slices of Subject 9 (35.6 weeks).
