# Supplementary material for: Rapid, High-resolution and Distortion-free R2* Mapping of Fetal Brain using Multi-echo Radial FLASH and Model-based Reconstruction
Source: ArXiv. 2025 Jan 7:arXiv:2501.00256v2. Preprint. [Version 2] (PMC11722525)
Supplement: Supplementary file 1 [file NIHPP2501.00256v2-supplement-1.pdf]

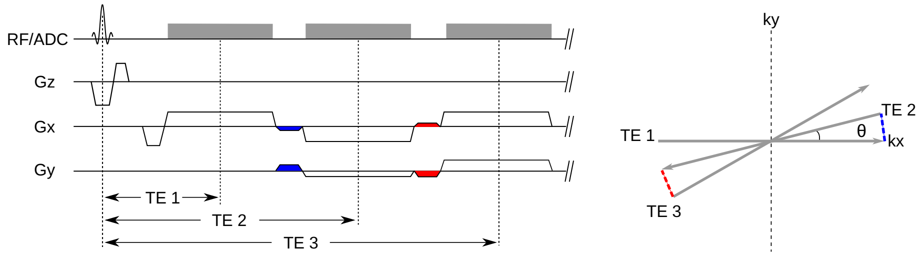

Supporting Information Figure S1. Schematic diagram of the 2D multi-echo radial FLASH sequence (three echoes are shown). Blip gradients (blue and red regions) are introduced among echoes to enable a complementary k-space coverage. θ is determined in a way that spokes from all echoes and 3 TRs are equally distributed. I.e., with 35 echoes and 3 TRs, $\theta = 360^\circ/(35 \times 3)$.

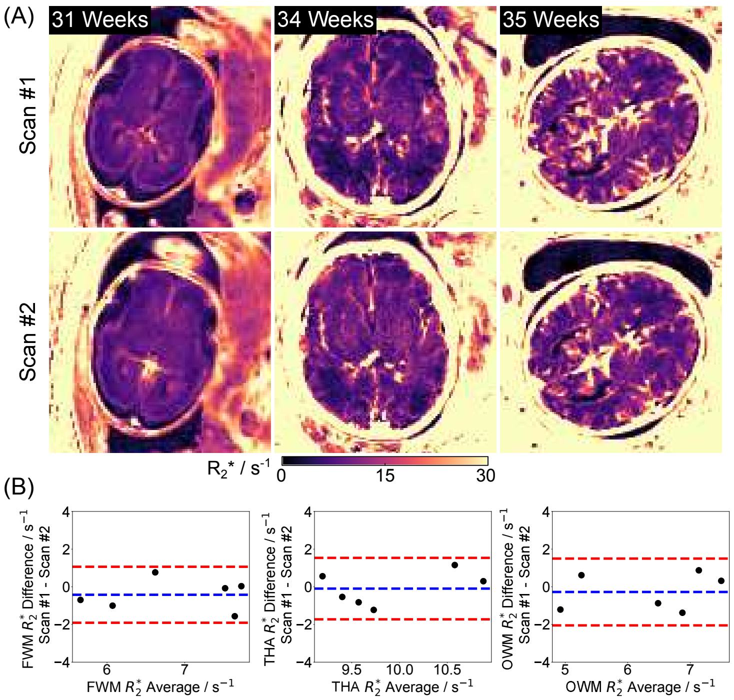

Supporting Information Figure S2. (A). Quantitative fetal brain R_2^* maps for two repeated scans across three subjects. (B). The scan-rescan R_2^* differences are -0.4 ± 0.8 s^{-1}, -0.1 ± 0.8 s^{-1} and -0.3 ± 0.9 s^{-1} for FWM, THA and OWM.

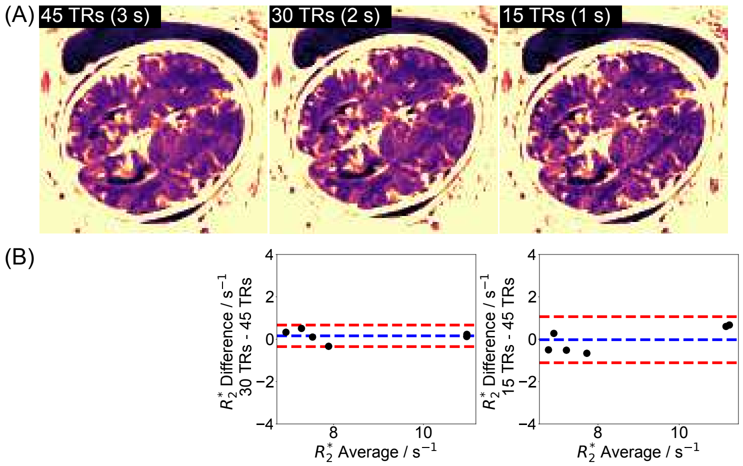

Supporting Information Figure S3. (A). Quantitative R_2^* maps for Subject 5 estimated using 3-second (45 TRs), 2-second (30 TRs), and 1-second (15 TRs) multi-echo radial FLASH acquisitions. Because of minimal motion during data acquisition, the 2-second and 1-second data were retrospectively undersampled from the 3-second one. (B) Bland-Altman plots comparing mean R_2^* values between the 3-second and 2-second, and 3-second and 1-second acquisitions. The mean difference are 0.16 ± 0.26 s^{-1} and -0.02 ± 0.6 s^{-1}, respectively.
